# Supplementary material for: Hot spot prediction in protein-protein interactions by an ensemble system
Source: BMC Syst Biol. 2018 Dec 31;12(Suppl 9):132. doi: 10.1186/s12918-018-0665-8 (PMC6311905; doi:10.1186/s12918-018-0665-8)
Supplement: Supplementary file 2 — All relevant MATLAB code. A simple Matlab implement of our predictor. (ZIP 19,742 kb) [file 12918_2018_665_MOESM2_ESM.zip › Additional file 2/Read ASA/ASA website/SKEMPI/1UUZA.htm]

 NetSurfP - Protein Surface Accessibility and Secondary Structure Predictions

  

|  |  |  |
| --- | --- | --- |
|  |  | NetSurfP - Protein Surface Accessibility and Secondary Structure Predictions  Technical University of Denmark |

  


---

```
# For publication of results, please cite:
# A generic method for assignment of reliability scores applied to solvent accessibility predictions.
# Bent Petersen, Thomas Nordahl Petersen, Pernille Andersen, Morten Nielsen and Claus Lundegaard
# BMC Structural Biology 2009, 9:51 doi:10.1186/1472-6807-9-51
#
# Column 1: Class assignment - B for buried or E for Exposed - Threshold: 25% exposure, but not based on RSA
# Column 2: Amino acid
# Column 3: Sequence name
# Column 4: Amino acid number
# Column 5: Relative Surface Accessibility - RSA
# Column 6: Absolute Surface Accessibility
# Column 7: Z-fit score for RSA prediction
# Column 8: Probability for Alpha-Helix
# Column 9: Probability for Beta-strand
# Column 10: Probability for Coil
E E  1UUZA                  1    0.892 155.867   1.085   0.003   0.003   0.994
E E  1UUZA                  2    0.598 104.401  -0.893   0.058   0.017   0.925
E Q  1UUZA                  3    0.524  93.497  -0.209   0.181   0.016   0.803
B P  1UUZA                  4    0.266  37.802  -1.070   0.339   0.016   0.645
E R  1UUZA                  5    0.373  85.532   0.341   0.522   0.016   0.462
B L  1UUZA                  6    0.141  25.854   0.272   0.802   0.014   0.185
B F  1UUZA                  7    0.305  61.274  -0.509   0.802   0.014   0.185
E E  1UUZA                  8    0.470  82.179   0.239   0.802   0.014   0.185
B L  1UUZA                  9    0.086  15.820  -0.076   0.802   0.014   0.185
B L  1UUZA                 10    0.163  29.790  -0.909   0.600   0.003   0.397
E G  1UUZA                 11    0.570  44.875  -0.605   0.246   0.004   0.750
E Q  1UUZA                 12    0.376  67.225   0.252   0.053   0.005   0.942
E P  1UUZA                 13    0.533  75.562  -1.344   0.246   0.004   0.750
E G  1UUZA                 14    0.535  42.128  -1.055   0.257   0.016   0.727
B Y  1UUZA                 15    0.145  31.029   0.248   0.522   0.016   0.462
E K  1UUZA                 16    0.314  64.652   0.928   0.938   0.007   0.055
E A  1UUZA                 17    0.515  56.731   0.612   0.923   0.002   0.076
B T  1UUZA                 18    0.173  24.037   0.676   0.970   0.001   0.030
B W  1UUZA                 19    0.048  11.448   0.995   0.970   0.001   0.030
E H  1UUZA                 20    0.381  69.340   1.565   0.970   0.001   0.030
E A  1UUZA                 21    0.523  57.624   1.242   0.970   0.001   0.030
B M  1UUZA                 22    0.078  15.668   0.507   0.923   0.002   0.076
B F  1UUZA                 23    0.148  29.744   0.285   0.858   0.002   0.139
E K  1UUZA                 24    0.627 128.892   1.434   0.600   0.003   0.397
E G  1UUZA                 25    0.560  44.048  -0.902   0.321   0.003   0.675
E E  1UUZA                 26    0.334  58.280  -0.089   0.321   0.003   0.675
E S  1UUZA                 27    0.561  65.784   0.822   0.430   0.016   0.555
E D  1UUZA                 28    0.454  65.421   1.114   0.257   0.016   0.727
B V  1UUZA                 29    0.047   7.239   0.783   0.321   0.003   0.675
B P  1UUZA                 30    0.168  23.768   0.154   0.502   0.002   0.495
E K  1UUZA                 31    0.496 102.027   1.303   0.858   0.002   0.139
B W  1UUZA                 32    0.087  20.851   0.150   0.879   0.010   0.111
B V  1UUZA                 33    0.046   7.086   0.170   0.879   0.010   0.111
E S  1UUZA                 34    0.401  47.032   0.572   0.802   0.014   0.185
E D  1UUZA                 35    0.517  74.543   0.365   0.622   0.015   0.363
B A  1UUZA                 36    0.296  32.575  -1.272   0.181   0.016   0.803
E S  1UUZA                 37    0.451  52.834  -1.513   0.058   0.017   0.925
E G  1UUZA                 38    0.338  26.585  -1.678   0.053   0.043   0.903
B P  1UUZA                 39    0.258  36.596  -1.504   0.052   0.084   0.864
B S  1UUZA                 40    0.295  34.609  -1.822   0.056   0.142   0.802
B S  1UUZA                 41    0.275  32.277  -0.455   0.018   0.088   0.893
E P  1UUZA                 42    0.287  40.739  -0.522   0.052   0.084   0.864
B S  1UUZA                 43    0.227  26.616  -1.107   0.056   0.142   0.802
E T  1UUZA                 44    0.416  57.699  -0.070   0.069   0.386   0.545
E S  1UUZA                 45    0.373  43.657  -0.021   0.023   0.655   0.322
B L  1UUZA                 46    0.148  27.099   0.529   0.021   0.756   0.223
E S  1UUZA                 47    0.415  48.615  -0.502   0.018   0.846   0.136
E L  1UUZA                 48    0.286  52.422  -0.259   0.021   0.756   0.223
E E  1UUZA                 49    0.671 117.189  -0.222   0.004   0.085   0.910
E G  1UUZA                 50    0.581  45.717  -1.179   0.005   0.015   0.979
E Q  1UUZA                 51    0.500  89.371   0.619   0.022   0.359   0.619
E P  1UUZA                 52    0.356  50.587   0.836   0.003   0.718   0.279
B Y  1UUZA                 53    0.083  17.823   1.122   0.011   0.918   0.071
B V  1UUZA                 54    0.134  20.580   0.898   0.011   0.918   0.071
B L  1UUZA                 55    0.040   7.379  -0.601   0.011   0.918   0.071
B A  1UUZA                 56    0.051   5.565  -0.016   0.018   0.846   0.136
B N  1UUZA                 57    0.201  29.485  -0.637   0.023   0.655   0.322
B S  1UUZA                 58    0.074   8.649  -0.798   0.069   0.386   0.545
B C  1UUZA                 59    0.042   5.897  -0.150   0.066   0.296   0.638
E K  1UUZA                 60    0.338  69.485   0.988   0.064   0.216   0.721
E P  1UUZA                 61    0.339  48.104  -0.513   0.052   0.084   0.864
B H  1UUZA                 62    0.236  42.910  -0.232   0.018   0.047   0.935
E D  1UUZA                 63    0.362  52.107  -0.817   0.018   0.019   0.964
B C  1UUZA                 64    0.060   8.410  -0.723   0.058   0.017   0.925
E G  1UUZA                 65    0.292  22.949  -0.377   0.339   0.016   0.645
B N  1UUZA                 66    0.259  37.859  -1.182   0.430   0.016   0.555
B N  1UUZA                 67    0.260  38.108   0.452   0.502   0.102   0.396
B R  1UUZA                 68    0.185  42.388   0.524   0.478   0.309   0.214
B L  1UUZA                 69    0.038   6.903  -0.009   0.391   0.466   0.143
B L  1UUZA                 70    0.045   8.166   0.601   0.273   0.587   0.140
B V  1UUZA                 71    0.025   3.812   0.563   0.265   0.667   0.069
B A  1UUZA                 72    0.042   4.595   0.291   0.157   0.773   0.069
B F  1UUZA                 73    0.054  10.938   0.619   0.085   0.775   0.140
B R  1UUZA                 74    0.158  36.159   0.499   0.021   0.451   0.528
E G  1UUZA                 75    0.489  38.516  -0.161   0.018   0.088   0.893
E D  1UUZA                 76    0.727 104.790   0.570   0.005   0.015   0.979
E K  1UUZA                 77    0.523 107.499  -0.111   0.018   0.019   0.964
E S  1UUZA                 78    0.519  60.792  -0.265   0.005   0.045   0.951
E A  1UUZA                 79    0.365  40.234   1.107   0.004   0.514   0.481
B A  1UUZA                 80    0.048   5.246  -0.174   0.002   0.816   0.182
B Y  1UUZA                 81    0.075  16.070   0.525   0.001   0.900   0.099
B G  1UUZA                 82    0.030   2.337  -1.022   0.001   0.900   0.099
B L  1UUZA                 83    0.041   7.525   0.453   0.001   0.959   0.040
B Q  1UUZA                 84    0.057  10.127   0.607   0.001   0.959   0.040
B V  1UUZA                 85    0.052   7.916   0.083   0.001   0.959   0.040
B S  1UUZA                 86    0.182  21.366  -0.086   0.001   0.900   0.099
B L  1UUZA                 87    0.081  14.849   0.853   0.004   0.514   0.481
E P  1UUZA                 88    0.467  66.253   0.744   0.004   0.197   0.799
E D  1UUZA                 89    0.607  87.469  -0.725   0.018   0.019   0.964
E E  1UUZA                 90    0.628 109.781  -0.966   0.018   0.019   0.964
E P  1UUZA                 91    0.551  78.130  -1.691   0.058   0.017   0.925
B A  1UUZA                 92    0.254  27.947  -0.796   0.115   0.016   0.868
E E  1UUZA                 93    0.571  99.736  -0.869   0.184   0.043   0.773
E V  1UUZA                 94    0.377  57.960  -0.974   0.191   0.086   0.723
B M  1UUZA                 95    0.214  42.841  -0.866   0.199   0.152   0.649
E Q  1UUZA                 96    0.521  93.033  -0.686   0.113   0.087   0.800
E T  1UUZA                 97    0.380  52.706  -0.504   0.053   0.043   0.903
B P  1UUZA                 98    0.280  39.689  -0.773   0.113   0.043   0.844
E S  1UUZA                 99    0.358  41.911  -0.968   0.113   0.087   0.800
E K  1UUZA                100    0.429  88.286   0.215   0.118   0.150   0.732
B Y  1UUZA                101    0.236  50.412  -0.686   0.135   0.317   0.548
B A  1UUZA                102    0.066   7.251  -0.349   0.069   0.386   0.545
E T  1UUZA                103    0.336  46.589   0.623   0.169   0.612   0.219
B Y  1UUZA                104    0.091  19.361   0.858   0.087   0.683   0.230
B R  1UUZA                105    0.279  63.845   0.843   0.172   0.690   0.138
B W  1UUZA                106    0.094  22.559  -0.313   0.159   0.516   0.325
B Y  1UUZA                107    0.148  31.585   0.147   0.064   0.216   0.721
E G  1UUZA                108    0.345  27.136  -1.244   0.004   0.085   0.910
E E  1UUZA                109    0.415  72.466   0.889   0.005   0.015   0.979
E P  1UUZA                110    0.276  39.150  -0.355   0.005   0.015   0.979
E S  1UUZA                111    0.421  49.353   0.155   0.176   0.004   0.820
E R  1UUZA                112    0.768 175.941   1.021   0.923   0.002   0.076
E Q  1UUZA                113    0.446  79.638   0.582   0.923   0.002   0.076
B V  1UUZA                114    0.039   6.040  -0.267   0.970   0.001   0.030
E R  1UUZA                115    0.296  67.853   1.588   0.970   0.001   0.030
E E  1UUZA                116    0.448  78.213   1.655   0.970   0.001   0.030
B L  1UUZA                117    0.118  21.569   1.366   0.970   0.001   0.030
B L  1UUZA                118    0.029   5.383   0.705   0.970   0.001   0.030
E M  1UUZA                119    0.285  57.008   1.499   0.970   0.001   0.030
E K  1UUZA                120    0.501 103.138   1.591   0.970   0.001   0.030
B Q  1UUZA                121    0.120  21.503   0.282   0.970   0.001   0.030
B L  1UUZA                122    0.073  13.403   0.447   0.923   0.002   0.076
E E  1UUZA                123    0.511  89.289   0.703   0.782   0.003   0.216
E S  1UUZA                124    0.762  89.342   0.966   0.502   0.002   0.495
E D  1UUZA                125    0.255  36.745   0.636   0.033   0.001   0.966
E P  1UUZA                126    0.568  80.542  -0.984   0.016   0.005   0.979
E N  1UUZA                127    0.585  85.659   0.006   0.003   0.003   0.994
B W  1UUZA                128    0.173  41.606  -0.519   0.005   0.015   0.979
E K  1UUZA                129    0.588 120.910   0.647   0.018   0.019   0.964
E L  1UUZA                130    0.675 123.629  -0.785   0.003   0.003   0.994

Explain the output.  Go back.
```

---
